# Acute development of syringomyelia following TBM in a pediatric case

**DOI:** 10.1186/s12887-021-02493-7

**Published:** 2021-01-14

**Authors:** Ningyuan Wen, Fumin Zhao, Yu Zhu, Fenglin Jia, Chaomin Wan, Yang Wen

**Affiliations:** 1 College of Clinical Medicine, Sichuan University, Chengdu, China; 2 Department of Radiology, West China Second University Hospital, Sichuan University, Chengdu, China; 3 Key Laboratory of Birth Defects and Related Diseases of Women and Children (Sichuan University), Ministry of Education, Chengdu, China; 4 Department of Pediatrics, West China Second University Hospital, Sichuan University, No. 20, 3rd Section of Renmin South Road, 610041 Chengdu, China

**Keywords:** Extrapulmonary tuberculosis, Tuberculous radiculomyelopathy, Paraplegia

## Abstract

**Background:**

Syringomyelia secondary to tuberculous meningitis (TBM) is rarely reported, and is usually a late complication. Acute development of syringomyelia following TBM is an exceedingly rare condition with only a few cases published in adults and no previous reports in children.

**Case presentation:**

We present a case of syringomyelia as an acute complication of TBM in a 12-year-old boy despite appropriate chemotherapy. The patient developed spastic paraplegia of the lower limbs with fecal and urinary retention seventeen days after the initial symptoms of TBM. He was managed successfully with continued chemotherapy and high-dose intravenous immunoglobulin (IVIG).

**Conclusions:**

This case reminds us that syrinx formation may be responsible for early neurological deterioration in children being managed for TBM. IVIG may be considered as an effective treatment option for this situation.

## Background

Tuberculosis (TB) still remains an important issue for global public health despite the decrease of medical burden caused by the disease. A report by the World Health Organization says that with 10 million TB patients and 1.5 million TB-related deaths, it remains one of the top infectious killers worldwide [1]. Among these cases, tuberculosis of central nervous system (CNS) is characterized by high risk of mortality and disability. Tuberculous meningitis (TBM) is one major form of CNS tuberculosis, while spinal involvement of TB is less well recognized. Syringomyelia is defined as the abnormal cavitation of the spinal cord. Cases of syringomyelia secondary to TBM are rarely reported, and is usually a late complication of TBM which may not occur until several years of the acute infection. Acute development of syringomyelia following TBM is an exceedingly rare condition with only a few cases published in adults and no previous reports in children. Currently, no drugs are proven effective to treat spinal cord complications of TBM including syringomyelia. Hereon, we report a 12-year-old boy who presented acute-onset generalized arachnoiditis and syringomyelia complicating TBM. High-dose intravenous immunoglobulin (IVIG) was used to treat syringomyelia complicating TBM and seemed to be effective.

## Case presentation

A 12-year-old boy was admitted to West China Second University Hospital, Chengdu on 13th April 2019 with the chief complaints of low-grade fever, cough for over a month, and headache, vomiting, progressive disturbance of consciousness for 3 weeks. He suffered from several generalized seizures throughout the course of the illness. His parents reported that he was previously healthy. There was no known contact with an individual with active tuberculosis although they resided in Tibet, where tuberculosis is highly prevalent. Vaccination status with Bacillus Calmette-Guerin was unknown.

On examination, his temperature was 37.8 °C, heart rate 74/min and respiration rate 20/min. No BCG scar was observed on his upper arms. The patient was confused and lethargic, and had neck stiffness and positive Kernig’s sign. The lower limbs had normal muscle tone, and can be lifted off the bed when giving pain stimulation. The Babinski sign was absent. There were crackles in the left lung.

Blood tests showed white blood cell 7.6×10^9^/L, absolute value of neutrophils 5.3×10^9^/L, hemoglobin level of 140 g/L, and platelet count 223 ×10^9^/L. The C-reactive protein was less than 0.8 mg/L. Liver and kidney function tests were normal. A tuberculin skin test was negative, while the interferon-gamma release assay was positive. Several sputum acid-fast bacilli stains, and an induced sputum culture for Mycobacterium tuberculosis (MTB) were negative. Contrast-enhanced chest computed tomography (CT) demonstrated consolidation in the apical posterior segment of the left upper lobe with hilar and mediastinal lymphadenopathy. Contrast-enhanced CT of the brain revealed dilated lateral ventricles and basilar meningeal enhancement. Analysis of cerebrospinal fluid (CSF) examination revealed a white blood count of 569×10^6^/L (80% lymphocytes), a remarkably elevated level of protein (4.52 g/L) and adenosine deaminase (89.4 U/L), as well as a decreased level of glucose (1.84 mmol/L) and chlorides (86.7 mmol/L). Xpert MTB/RIF test on bronchoalveolar lavage fluid demonstrated a diagnosis of rifampicin-sensitive tuberculosis.

The patient was diagnosed with pulmonary TB and TBM and commenced on standard anti-tubercular therapy (isoniazid, rifampicin, pyrazinamide and ethambutol) with dexamethasone 0.6 mg/kg.d. In the following days, the patient became conscious and oriented, and experienced no recurrence of seizure or vomiting. Other symptoms including fever and headache were gradually relieved. However, seventeen days after treatment, he developed right upper quadrant pain and lumbodorsal pain, and thereafter, he developed spastic paraplegia of the lower limbs with fecal and urinary retention. On examination, there was motor weakness in the lower extremity (manual muscle testing assessed 1/5) as well as impaired sensation in right lower limb. Hypertonia and hyperreflexia were detected in the lower limbs. Repeat routine blood test and C-reactive protein were normal. MRI of the spine demonstrated generalized inflammation of spinal cord and meninges with enlarged syringomyelic cavities. Syrinx formation was exceptionally notable from C1 to T1 with the maximum width of 5.9 mm in the central canal of the cervical cord (Fig. 1a). T2-weighted images showed hyperintense signals within the spinal cord parenchyma (Fig. 1b). Contrast enhanced MRI demonstrated significant thickening and enhancement of the arachnoid, with narrow and even disappeared subarachnoid space (Fig. 1c). Hyperintense signals on T2 and isointense signals on T1-weighted images were shown in the subarachnoid space. There was atrophy of spinal cord from T6 to T8 (Figure 1c), and nodular enhancement of conus medullaris was shown (Fig. 1d).

**Fig.**
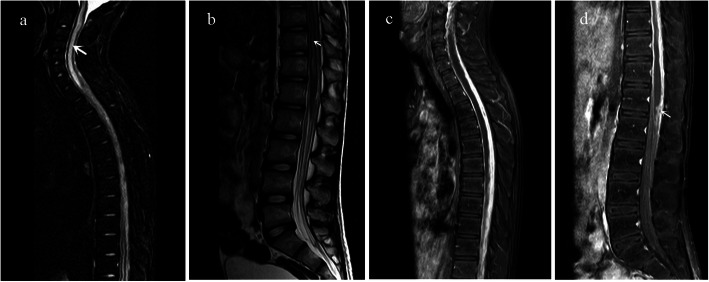
**a** Sagittal fat-suppressed T2-weighted MRI scan of the spine demonstrating notable dilation of the central canal of the cervical cord (Syrinx formation) from C1 to T1 (arrow). **b** Sagittal T2-weighted image of the spine demonstrating hyperintense signals within the lumbar cord (arrow) and conus medullaris. **c** Sagittal post-contrast T1-weighted image demonstrating extensive thickening and enhancement of the arachnoid, disappeared subarachnoid space, and atrophy of thoracic spinal cord. **d** Sagittal post-contrast T1-weighted image demonstrating nodular enhancement of conus medullaris (arrow)

The clinical deterioration and the spinal involvement were unexpected since the patient was receiving effective anti-tubercular treatment with initial improvement. After discussion with the pediatric neurosurgeon, surgery was believed to be unrewarding given the extensive arachnoiditis with no obvious cord compression. The development of spinal cord complications was thought to be caused by the exaggerated cell-mediated immune response against mycobacterial antigens, while other etiologies, like superimposed bacterial infection and drug-resistant TB were thought less likely. High-dose intravenous immunoglobulin (IVIG, 2 g/kg) was given to the patient given the progressive deterioration of the symptoms and potential efficacy of IVIG as an immunoregulatory and anti-inflammatory drug. The patient responded well and went on for a steady recovery. Seven days after the use of IVIG together with anti-tubercular drugs and dexamethasone, the patient was not complaining of any urinary disturbance, back pain, headache or vomiting. Manual muscle testing assessed 4/5 in his lower extremities and he was able to walk under assistance. Repeat MRI showed radiological improvement of syringomyelia and other spinal lesions. He was then discharged for further rehabilitation at a local hospital.

## Discussion and conclusions

Spinal cord and spinal nerve root involvement is a common yet frequently overlooked complication in the setting of TBM. It is estimated that around 10% of TBM cases have some form of spinal cord involvement [2]. The most common form of spinal cord involvement is tuberculous radiculomyelopathy, which accounts for 38.7% of TBM complications [3]. Other complications following TBM include spinal tuberculoma, myelitis, syringomyelia and spinal tuberculous abscess. Spinal cord involvement in TBM is described in the literature as case reports or small case series. High CSF level of protein is a risk factor for tuberculous arachnoiditis. Syringomyelia complicating TBM, however, is a rare condition, which is believed to be associated with worse prognosis [4]. Pathologically, syringomyelia is defined as a condition in which cavities filled with fluid developing in central canal of spinal cord [5]. As an accompaniment of TBM, formation of spinal cord syrinx often takes several years after patients have recovered from the original TBM onset. The maximum reported latent period between the initial inflammatory event and that of syringomyelia is 30 years [3]. This kind of complication may occur paradoxically when the patient is still receiving effective antitubercular therapy. The exact pathogenesis of syrinx formation remains unknown, and a few mechanisms have been proposed. One hypothesis is that an obliterative endarteritis causing ischemic injury or softening of spinal cord leads to cavitation in spinal cord parenchyma. Alternatively, focal meningeal and spinal parenchymal scarring may lead to obstruction of the CSF flow, thus pushing CSF into the central canal through Virchow-Robin spaces [3]. The latter hypothesis is supported by our case as arachnoiditis had vanished the subarachnoid space.

Typical clinical manifestations of syringomyelia include progressive spastic paraparesis and sphincter disturbance. MRI plays an essential role in terms of its diagnosis, as it clearly demonstrates features of syrinx cysts as well as other associated disorders [6]. Therapeutically, there is no specific therapy for syringomyelia apart from antitubercular drugs and corticosteroids. Corticosteroids are routinely used in TBM patients since they reduce mortality and minimize residual neurological deficits. High-dose corticosteroids were reported effective in patients with spinal cord involvement following TBM despite its unclear mechanism and lack of high-level evidence [7]. As a treatment option, surgical procedures are considered in deteriorating patients regardless of active medical treatment, including incision decompression and various kinds of shunting procedures. However, syringomyelia is usually accompanied by extensive arachnoid adhesions and surgical attempts generally fail.

In our case, the patient developed syringomyelia seventeen days after the initial diagnosis of TBM, suggesting an acute course, which is exceptionally rare as there have only been few reports identifying syringomyelia as an early complication of TBM [8–11]. The features of these previous cases are summarized in Table 1. All five patients suffered from certain degree of limb paralysis or sphincter dysfunction. Syrinxes were located in various segments of spinal cord, mostly the cervical or thoracic spinal cord [4]. By comparison, this is the first pediatric case in which syringomyelia occurs in an acute pattern secondary to TBM, and the first case that applied IVIG as part of the drug therapy. Considering the satisfactory outcome of this patient, the role of IVIG in such cases could be further discussed.

In conclusion, syringomyelia is a rare but noteworthy complication of TBM that lacks systematic research. This case reminds us that syrinx formation may be responsible for early neurological deterioration in children being managed for TBM. The MRI findings of the spine are helpful for a rapid diagnosis. Integration of the existing data from sporadic reports could be a meaningful effort to further discuss the etiology and treatment of the disease.

**Table 1:** Summary of features of TBM patients with syringomyelia as an early complication

Authors	Year	Region	Age	Time to onset of syringomyelia	Syrinx location	Treatment	Outcome
Daif et al. [8]	1997	Saudi Arabia	35	59 days	T_5_ to T_10_	Antitubercular drugs and steroids	lost to follow-up (prognosis not mentioned)
Daif et al. [8]	1997	Saudi Arabia	32	11 days	T_10_ to L_1_	Antitubercular drugs and steroids	Motor power improved but still incontinent
Moghtader et al. [9]	2006	Iran	27	Within 5 days	A 3–4 cm cavity in the lower part of thoracolumbar cord	Antitubercular drugs and Surgical exploration	General condition improved but motor power still impaired
Pandey et al. [10]	2013	India	26	90 days	C_3_ to T_3_	antitubercular drugs, steroids, antiedema drugs, phenytoin and thecoperitoneal shunt	Improved motor power
Ratre et al. [11]	2018	India	20	67 days	Cervico-medullary junction to T_10_	Antitubercular drugs and steroids, ventriculo-peritoneal shunt	No improvement, lost to follow-up

## Data Availability

The authors declare that deidentified data will be shared in terms of all available clinical examinations of the patient we report. Once the manuscript is published, readers are welcome to contact the corresponding author for data sharing.

## Abbreviations

TBM: Tuberculous meningitis
MRI: Magnetic resonance imaging
IVIG: Intravenous immunoglobulin
TB: Tuberculosis
CNS: Central nervous system
MTB: Mycobacterium tuberculosis
CT: Computed tomography
CSF: Cerebrospinal fluid
